# Supplementary material for: Quality Assessment of Three Types of Drinking Water Sources in Guinea-Bissau
Source: Int J Environ Res Public Health. 2020 Oct 4;17(19):7254. doi: 10.3390/ijerph17197254 (PMC7579607; doi:10.3390/ijerph17197254)
Supplement: Supplementary file 1 [file ijerph-17-07254-s001.pdf]

Supplementary. Raw data tables

Table S1: Annex 1. The raw data used for physicochemical analysis. Footnote references is added below table.

Year	Season	Location	City	Sampling point	pH	T	Sal	Turb	EC	ORP	DO	TDS	NO2−	NO3-	Cr (VI)	Fe2+	SO_4^{2-}	P	Alk	Cu	SO_3^{2-}	Hardness	Residual chlorine
								(UNT)	(µs/cm2)	mv	(mg/l)	(mg/l)	0-0.5 mg/l N	0-1 mg/l N	0-1 mg/l	0-10 mg/l	0-200 mg/l	0-100 mg/l	0 -500 mg/l CaCO3	0-5 mg/l	0-500 mg/l Na2SO 3	0-500 mg/l CaCO3	mg/l
2019	**Dry**	H3A	Bissau	Hole	8.60	30.10	0.26	0.58	530.00	201.60	1.25	185.00	0.01	0.36	0.12	0.00	0.00	34.70	205.00	0.04	14.00	0.00	0.10
2019	**Dry**	H3A	Bissau	Hole	8.20	30.00	0.26	0.59	530.00	202.00	1.27	187.00	0.01	0.37	0.13	0.00	0.00	34.63	204.80	0.05	13.60	0.00	0.11
2019	**Dry**	H3A	Bissau	Hole	8.10	29.58	0.26	0.61	525.00	199.00	1.19	182.00	0.01	0.40	0.15	0.00	0.00	35.00	209.00	0.05	15.20	0.00	0.10
2019	**Dry**	H3A	Bissau	Hole	8.15	31.00	0.26	0.65	534.00	205.00	1.26	189.00	0.01	0.39	0.15	0.00	0.00	32.45	208.98	0.05	14.23	0.00	0.12
2019	**Dry**	H3A	Bissau	Hole	8.55	30.00	0.26	0.59	529.00	200.00	1.20	181.96	0.01	0.36	0.14	0.00	0.00	34.10	205.16	0.04	13.69	0.00	0.10
2019	**Dry**	H3A	Bissau	Hole	8.60	31.00	0.26	0.58	526.00	196.00	1.17	179.68	0.01	0.38	0.12	0.00	0.00	34.85	206.70	0.05	14.10	0.00	0.10
2019	**Dry**	H3A	Bissau	Tap	8.58	30.90	0.26	0.48	525.00	151.40	3.25	186.00	0.01	0.50	0.08	0.00	0.00	24.00	205.00	0.06	17.00	10.00	0.02
2019	**Dry**	H3A	Bissau	Tap	8.50	30.81	0.26	0.46	522.00	150.48	3.27	186.79	0.00	0.49	0.09	0.00	0.00	23.95	205.00	0.05	16.56	9.68	0.01
2019	**Dry**	H3A	Bissau	Tap	8.49	30.10	0.26	0.50	532.00	162.00	3.38	187.32	0.00	0.52	0.08	0.00	0.00	25.20	219.03	0.07	17.00	10.24	0.02
2019	**Dry**	H3A	Bissau	Tap	7.98	31.00	0.26	0.51	498.00	159.00	3.10	184.65	0.01	0.51	0.07	0.00	0.00	24.52	199.78	0.07	17.20	10.78	0.02
2019	**Dry**	H3A	Bissau	Tap	8.62	29.00	0.26	0.49	502.00	149.00	3.00	189.21	0.01	0.49	0.08	0.00	0.00	23.89	204.85	0.06	15.87	9.87	0.01
2019	**Dry**	H3A	Bissau	Tap	8.59	31.00	0.26	0.52	509.00	152.63	3.10	186.02	0.00	0.53	0.07	0.00	0.00	23.09	205.36	0.07	16.45	11.20	0.02
2019	**Dry**	H3A	Bissau	Fountain	8.59	29.60	0.26	0.98	523.00	150.80	3.56	184.00	0.00	0.82	0.12	0.10	0.00	21.60	205.00	0.06	17.00	10.00	0.01
2019	**Dry**	H3A	Bissau	Fountain	8.59	29.00	0.25	0.10	520.00	148.00	3.50	179.00	0.01	0.81	0.12	0.10	0.00	21.00	204.39	0.06	16.98	9.00	0.01
2019	**Dry**	H3A	Bissau	Fountain	8.48	28.94	0.24	0.10	531.00	152.02	3.42	180.98	0.00	0.81	0.13	0.09	0.00	21.58	206.30	0.06	17.04	10.00	0.00
2019	**Dry**	H3A	Bissau	Fountain	8.45	30.00	0.26	0.11	524.30	147.64	3.20	178.56	0.01	0.83	0.12	0.10	0.00	20.89	205.47	0.06	17.98	10.00	0.01
2019	**Dry**	H3A	Bissau	Fountain	8.29	29.24	0.26	0.10	529.01	150.10	3.50	185.23	0.01	0.82	0.13	0.10	0.00	22.10	203.89	0.07	16.58	10.00	0.01
2019	**Dry**	H3A	Bissau	Fountain	8.50	30.00	0.26	0.10	512.49	152.02	3.51	187.40	0.00	0.82	0.13	0.11	0.00	21.26	204.78	0.07	17.05	10.00	0.01
2019	**Dry**	HSE	Bissau	Hole	8.50	31.60	0.26	1.00	521.00	114.20	3.41	182.00	0.00	0.73	0.10	0.05	0.00	31.20	220.00	0.02	16.00	0.00	0.00
2019	**Dry**	HSE	Bissau	Hole	8.20	31.00	0.26	0.91	511.00	114.10	3.20	182.00	0.00	0.72	0.10	0.06	0.00	31.11	210.00	0.03	16.00	0.00	0.00
2019	**Dry**	HSE	Bissau	Hole	8.10	30.00	0.26	1.00	529.00	119.20	3.42	183.90	0.00	0.73	0.09	0.06	0.00	32.00	223.00	0.02	15.23	0.00	0.00
2019	**Dry**	HSE	Bissau	Hole	8.48	31.28	0.26	0.95	519.56	120.00	3.39	184.00	0.00	0.71	0.10	0.06	0.00	31.89	218.00	0.03	17.20	0.00	0.00
2019	**Dry**	HSE	Bissau	Hole	8.50	31.45	0.26	0.98	528.07	111.00	3.21	182.32	0.00	0.73	0.10	0.05	0.00	32.05	222.00	0.02	16.45	0.00	0.00

2019	**Dry**	HSE	Bissau	Hole	8.39	30.49	0.26	1.00	530.14	113.96	3.52	179.89	0.00	0.73	0.10	0.06	0.00	31.75	221.89	0.02	17.00	0.00	0.00
2019	**Dry**	HSE	Bissau	Reservoir outlet	8.52	31.40	0.26	0.79	522.00	107.50	3.11	179.00	0.01	1.00	0.06	0.00	0.00	24.00	225.00	0.04	16.00	0.00	0.02
2019	**Dry**	HSE	Bissau	Reservoir outlet	8.51	31.39	0.26	0.80	522.00	107.10	3.00	179.00	0.01	1.00	0.06	0.00	0.00	24.00	224.90	0.04	15.00	0.00	0.02
2019	**Dry**	HSE	Bissau	Reservoir outlet	8.49	31.00	0.26	0.81	520.98	105.26	3.12	179.85	0.01	0.98	0.05	0.00	0.00	23.18	220.98	0.03	17.02	0.00	0.02
2019	**Dry**	HSE	Bissau	Reservoir outlet	8.53	29.25	0.26	0.83	519.20	105.12	2.98	176.85	0.01	0.99	0.06	0.00	0.00	23.89	199.89	0.04	15.89	0.00	0.03
2019	**Dry**	HSE	Bissau	Reservoir outlet	8.14	32.00	0.26	0.82	523.00	109.20	2.96	176.25	0.01	1.00	0.04	0.00	0.00	25.10	212.58	0.05	16.45	0.00	0.01
2019	**Dry**	HSE	Bissau	Reservoir outlet	8.54	31.89	0.26	0.85	523.52	109.78	3.21	176.87	0.01	1.00	0.06	0.00	0.00	24.23	216.98	0.04	14.89	0.00	0.03
2019	**Dry**	HSE	Bissau	Fountain	8.46	31.30	0.26	0.81	519.00	113.30	3.20	182.00	0.00	0.72	0.10	0.00	0.00	31.30	225.00	0.04	17.00	0.00	0.00
2019	**Dry**	HSE	Bissau	Fountain	8.45	31.25	0.27	0.89	520.00	113.00	2.99	181.00	0.00	0.72	0.10	0.00	0.00	31.30	225.00	0.05	17.00	0.00	0.00
2019	**Dry**	HSE	Bissau	Fountain	8.38	30.00	0.26	0.90	521.00	110.39	3.19	178.00	0.00	0.73	0.10	0.00	0.00	32.00	223.58	0.06	17.45	0.00	0.00
2019	**Dry**	HSE	Bissau	Fountain	8.40	31.57	0.25	0.99	519.00	99.89	3.17	175.79	0.00	0.72	0.09	0.00	0.00	32.00	223.79	0.05	17.58	0.00	0.00
2019	**Dry**	HSE	Bissau	Fountain	8.48	31.02	0.26	0.82	520.00	98.46	3.24	173.48	0.00	0.72	0.10	0.00	0.00	31.78	224.01	0.04	16.98	0.00	0.00
2019	**Dry**	HSE	Bissau	Fountain	8.45	31.00	0.25	0.89	521.56	116.85	3.16	175.00	0.00	0.73	0.10	0.00	0.00	32.24	225.00	0.05	17.72	0.00	0.00
2019	**Dry**	BLELA	Quinhamel	Shallow wells	6.35	27.50	0.04	12.00	167.00	175.20	2.00	125.00	0.02	4.00	0.12	0.15	3.00	0.10	30.00	0.10	15.00	20.00	0.15
2019	**Dry**	BLELA	Quinhamel	Shallow wells	6.40	27.40	0.04	13.00	167.58	175.00	3.00	126.00	0.02	4.00	0.12	0.15	4.00	0.10	30.00	0.10	14.00	19.45	0.16
2019	**Dry**	BLELA	Quinhamel	Shallow wells	6.30	28.00	0.05	15.00	158.00	175.35	3.00	124.85	0.02	4.00	0.13	0.15	4.00	0.09	29.58	0.10	15.00	20.23	0.16
2019	**Dry**	BLELA	Quinhamel	Shallow wells	6.29	27.42	0.04	13.00	170.00	176.00	2.00	126.25	0.02	3.00	0.12	0.14	4.00	0.10	30.12	0.10	14.29	21.48	0.15
2019	**Dry**	BLELA	Quinhamel	Shallow wells	6.20	27.26	0.05	15.00	168.23	173.98	3.00	125.40	0.02	4.00	0.12	0.16	3.00	0.10	30.21	0.10	15.24	20.00	0.16
2019	**Dry**	BLELA	Quinhamel	Shallow wells	6.25	27.85	0.04	15.32	171.20	174.89	3.00	126.00	0.02	3.00	0.13	0.16	4.00	0.10	30.00	0.10	14.58	20.86	0.16
2019	**Dry**	INTOZINHO8	Quinhamel	Shallow wells	5.90	27.60	0.05	15.00	183.00	180.00	4.70	77.00	0.03	6.35	0.09	0.20	4.00	0.10	20.00	0.06	14.00	20.00	0.11
2019	**Dry**	INTOZINHO8	Quinhamel	Shallow wells	5.90	27.20	0.05	14.26	179.00	179.00	4.60	76.00	0.04	6.40	0.10	0.21	3.00	0.09	19.20	0.05	13.45	20.00	0.12
2019	**Dry**	INTOZINHO8	Quinhamel	Shallow wells	5.00	27.00	0.04	15.23	182.32	181.23	5.00	78.00	0.04	6.23	0.10	0.20	4.00	0.10	20.23	0.07	13.98	20.00	0.11
2019	**Dry**	INTOZINHO8	Quinhamel	Shallow wells	4.98	27.52	0.04	14.28	183.46	180.47	3.98	77.59	0.03	6.78	0.09	0.20	3.78	0.09	19.48	0.06	14.25	20.00	0.11
2019	**Dry**	INTOZINHO8	Quinhamel	Shallow wells	5.10	26.89	0.05	15.24	178.98	179.89	4.56	78.23	0.03	6.35	0.10	0.21	3.00	0.10	21.19	0.05	13.46	20.00	0.12
2019	**Dry**	INTOZINHO8	Quinhamel	Shallow wells	4.59	27.62	0.05	15.36	181.46	181.46	4.78	77.21	0.03	6.25	0.10	0.21	3.00	0.10	20.18	0.05	14.12	20.00	0.12
2019	**Dry**	BOR9	Quinhamel	Shallow wells	5.30	27.10	0.04	14.29	132.00	196.00	6.02	54.00	0.01	5.93	0.10	0.25	3.00	0.11	15.00	0.02	10.00	15.00	0.14
2019	**Dry**	BOR9	Quinhamel	Shallow wells	5.29	27.10	0.05	14.98	131.00	195.00	6.98	53.78	0.01	5.94	0.10	0.26	3.20	0.12	15.00	0.02	10.00	15.00	0.13
2019	**Dry**	BOR9	Quinhamel	Shallow wells	5.00	27.00	0.06	15.20	129.89	194.89	6.70	54.12	0.01	6.00	0.10	0.25	3.00	0.12	16.00	0.02	10.00	15.00	0.14
2019	**Dry**	BOR9	Quinhamel	Shallow wells	4.98	26.89	0.06	16.00	132.48	195.45	6.23	53.89	0.01	5.78	0.10	0.27	3.10	0.12	15.00	0.02	10.00	15.00	0.13
2019	**Dry**	BOR9	Quinhamel	Shallow wells	5.12	27.10	0.04	15.89	132.00	193.89	6.02	54.75	0.01	5.98	0.10	0.26	2.98	0.12	16.52	0.02	10.00	15.00	0.14
2019	**Dry**	BOR9	Quinhamel	Shallow wells	5.31	26.89	0.04	16.23	130.00	194.79	6.89	54.78	0.01	5.78	0.10	0.26	3.00	0.12	15.00	0.02	10.00	15.00	0.14

2019	**Dry**	BOR10	Quinhamel	Shallow wells	5.32	27.50	0.03	12.00	87.00	202.00	7.29	38.00	0.01	1.59	0.14	0.20	2.00	0.14	25.00	0.08	12.00	17.00	0.10
2019	**Dry**	BOR10	Quinhamel	Shallow wells	5.33	28.00	0.04	13.25	130.00	200.00	7.00	37.90	0.01	1.60	0.14	0.19	1.80	0.14	24.70	0.09	11.98	17.00	0.10
2019	**Dry**	BOR10	Quinhamel	Shallow wells	5.00	29.00	0.04	12.79	128.67	199.87	6.89	37.00	0.01	1.70	0.14	0.21	2.00	0.14	25.00	0.09	12.25	17.00	0.10
2019	**Dry**	BOR10	Quinhamel	Shallow wells	5.12	28.00	0.04	14.02	134.20	201.12	7.30	37.00	0.01	1.71	0.14	0.20	2.00	0.14	25.35	1.00	12.12	16.78	0.10
2019	**Dry**	BOR10	Quinhamel	Shallow wells	4.98	28.02	0.04	14.05	133.25	200.00	7.01	38.20	0.01	1.62	0.14	0.20	1.79	0.14	25.47	0.08	12.00	17.00	0.10
2019	**Dry**	BOR10	Quinhamel	Shallow wells	5.10	28.36	0.04	15.03	130.00	203.41	7.05	37.68	0.01	1.60	0.14	0.19	2.12	0.14	24.89	0.09	12.00	17.00	0.10
2019	**Dry**	QUINHAMEL 11	Quinhamel	Tubewell	5.55	28.60	0.14	0.98	129.00	211.50	2.90	67.00	0.02	1.00	0.05	0.00	2.00	0.18	10.00	0.00	16.00	55.00	0.18
2019	**Dry**	QUINHAMEL 11	Quinhamel	Tubewell	5.32	27.80	0.15	0.96	128.00	211.50	2.90	67.00	0.02	0.98	0.05	0.00	1.90	0.17	9.90	0.00	16.00	54.00	0.18
2019	**Dry**	QUINHAMEL 11	Quinhamel	Tubewell	5.30	28.00	0.15	1.20	132.00	210.23	3.00	68.00	0.02	0.79	0.05	0.00	1.98	0.16	10.00	0.00	15.00	55.00	0.18
2019	**Dry**	QUINHAMEL 11	Quinhamel	Tubewell	5.23	28.00	0.15	1.00	130.36	211.48	2.92	68.00	0.02	1.78	0.05	0.00	1.96	0.16	10.00	0.00	16.00	55.00	0.18
2019	**Dry**	QUINHAMEL 11	Quinhamel	Tubewell	5.22	27.95	0.15	1.00	131.25	211.98	2.98	68.20	0.02	1.10	0.05	0.00	2.00	0.17	10.00	0.00	17.00	56.00	0.18
2019	**Dry**	QUINHAMEL 11	Quinhamel	Tubewell	5.00	27.89	0.15	1.00	131.02	212.00	3.00	67.20	0.02	0.69	0.05	0.00	2.00	0.17	9.89	0.00	17.00	56.00	0.18
2019	**Dry**	QUINHAMEL 12	Quinhamel	Tubewell	5.30	28.86	0.11	3.25	240.00	184.70	2.40	121.00	0.00	0.71	0.06	0.00	0.00	0.13	5.00	0.00	10.00	50.00	0.12
2019	**Dry**	QUINHAMEL 12	Quinhamel	Tubewell	5.29	28.00	0.10	3.20	240.00	183.79	2.40	121.00	0.00	0.70	0.07	0.00	0.00	0.13	6.00	0.00	11.00	51.00	0.11
2019	**Dry**	QUINHAMEL 12	Quinhamel	Tubewell	5.12	29.00	0.10	3.00	238.00	179.58	2.40	121.23	0.00	0.71	0.08	0.00	0.00	0.13	5.00	0.00	10.00	51.00	0.11
2019	**Dry**	QUINHAMEL 12	Quinhamel	Tubewell	5.32	28.00	0.10	3.00	241.00	179.89	2.40	121.47	0.00	0.71	0.08	0.00	0.00	0.13	5.00	0.00	9.89	51.23	0.10
2019	**Dry**	QUINHAMEL 12	Quinhamel	Tubewell	5.28	28.00	0.10	3.25	241.89	181.47	2.41	121.98	0.00	0.72	0.06	0.00	0.00	0.13	6.00	0.00	11.00	52.14	0.10
2019	**Dry**	QUINHAMEL 12	Quinhamel	Tubewell	5.28	28.45	0.10	3.19	240.58	182.40	2.40	121.00	0.00	0.71	0.07	0.00	0.00	0.13	5.00	0.00	11.00	52.78	0.11
2019	**Dry**	QUINHAMEL 13	Quinhamel	Tubewell	4.66	27.98	0.02	1.83	41.00	234.90	4.60	21.00	0.00	0.91	0.07	0.00	2.00	0.15	0.00	0.00	17.00	0.00	0.10
2019	**Dry**	QUINHAMEL 13	Quinhamel	Tubewell	4.89	28.00	0.03	1.79	39.00	229.41	5.00	26.00	0.00	1.00	0.06	0.00	1.92	0.16	0.00	0.00	15.00	0.00	0.10
2019	**Dry**	QUINHAMEL 13	Quinhamel	Tubewell	4.90	28.00	0.02	1.82	41.00	230.00	5.00	26.12	0.00	0.99	0.06	0.00	1.99	0.16	0.00	0.00	16.00	0.00	0.10
2019	**Dry**	QUINHAMEL 13	Quinhamel	Tubewell	4.58	28.00	0.02	1.83	40.00	231.25	4.89	26.36	0.00	1.00	0.06	0.00	1.95	0.16	0.00	0.00	16.78	0.00	0.10
2019	**Dry**	QUINHAMEL 13	Quinhamel	Tubewell	4.81	27.00	0.02	1.82	41.00	233.45	4.58	25.87	0.00	1.00	0.07	0.00	2.00	0.16	0.00	0.00	16.87	0.00	0.10
2019	**Dry**	QUINHAMEL 13	Quinhamel	Tubewell	4.83	27.00	0.01	1.82	41.00	233.10	4.79	25.77	0.00	1.00	0.07	0.00	2.00	0.15	0.00	0.00	15.99	0.00	0.10
2019	**Dry**	BAGDAD	Quinhamel	Tubewell	4.66	28.83	0.05	1.40	128.00	250.90	5.50	60.00	0.01	1.01	0.07	0.00	2.00	0.22	10.00	0.02	9.00	25.00	0.06
2019	**Dry**	BAGDAD	Quinhamel	Tubewell	4.50	28.10	0.06	1.50	125.79	249.20	6.30	68.00	0.01	0.97	0.07	0.00	2.00	0.19	9.00	0.03	10.00	26.00	0.05
2019	**Dry**	BAGDAD	Quinhamel	Tubewell	4.52	28.00	0.06	1.49	127.00	250.00	5.58	68.77	0.01	1.02	0.07	0.00	1.98	0.22	10.00	0.03	10.00	26.20	0.06
2019	**Dry**	BAGDAD	Quinhamel	Tubewell	4.67	28.12	0.06	1.47	127.23	248.98	6.12	68.68	0.01	1.00	0.06	0.00	1.96	0.22	10.00	0.03	10.00	26.00	0.06
2019	**Dry**	BAGDAD	Quinhamel	Tubewell	4.53	28.09	0.06	1.52	127.56	251.20	6.13	65.89	0.01	1.00	0.06	0.00	2.34	0.23	10.00	0.02	10.98	26.37	0.05
2019	**Dry**	BAGDAD	Quinhamel	Tubewell	4.49	28.00	0.06	1.50	127.75	251.39	6.20	66.00	0.01	0.98	0.06	0.00	2.00	0.23	9.00	0.02	9.97	26.00	0.06

2019	**Dry**	QUINHAMEL 15	Quinhamel	Tubewell	4.51	28.56	0.02	2.10	49.00	255.00	7.40	24.00	0.01	0.97	0.10	0.05	3.00	0.22	5.00	0.00	15.00	0.00	0.07
2019	**Dry**	QUINHAMEL 15	Quinhamel	Tubewell	4.61	29.00	0.02	2.00	50.00	253.00	7.20	26.00	0.00	1.10	0.09	0.05	3.10	0.25	6.00	0.00	16.00	0.00	0.08
2019	**Dry**	QUINHAMEL 15	Quinhamel	Tubewell	4.60	30.00	0.02	2.15	50.00	251.00	7.00	24.89	0.01	1.09	0.10	0.05	3.05	0.25	6.00	0.00	16.00	0.00	0.08
2019	**Dry**	QUINHAMEL 15	Quinhamel	Tubewell	4.59	29.23	0.02	2.12	50.24	252.36	7.10	24.78	0.00	1.00	0.10	0.06	3.00	0.25	6.00	0.00	15.79	0.00	0.08
2019	**Dry**	QUINHAMEL 15	Quinhamel	Tubewell	4.61	29.00	0.02	2.10	50.56	255.47	7.42	25.23	0.01	1.00	0.10	0.06	3.09	0.25	5.80	0.00	15.58	0.00	0.08
2019	**Dry**	QUINHAMEL 15	Quinhamel	Tubewell	4.63	28.46	0.02	2.10	50.00	255.79	7.41	25.12	0.01	1.00	0.10	0.05	3.10	0.25	6.00	0.00	16.00	0.00	0.07
2019	**Dry**	BANDIM	Bissau	Tap	7.67	28.80	0.25	0.74	542.00	69.50	5.00	257.00	0.01	0.83	0.10	0.15	3.00	0.38	210.00	0.00	16.00	10.00	0.21
2019	**Dry**	BANDIM	Bissau	Tap	7.10	28.10	0.25	0.71	539.00	71.00	5.00	249.00	0.01	0.79	0.09	0.17	2.98	0.41	200.00	0.00	17.10	9.80	0.20
2019	**Dry**	BANDIM	Bissau	Tap	7.63	28.00	0.25	0.73	540.00	70.23	4.98	256.00	0.01	0.84	0.10	0.15	3.12	0.40	209.32	0.00	17.00	10.10	0.20
2019	**Dry**	BANDIM	Bissau	Tap	7.63	28.74	0.25	0.73	541.00	70.89	4.58	256.89	0.01	0.85	0.10	0.15	3.10	0.40	200.89	0.00	17.00	10.23	0.20
2019	**Dry**	BANDIM	Bissau	Tap	7.18	28.23	0.25	0.74	538.98	71.26	5.00	254.78	0.01	0.80	0.10	0.15	2.89	0.41	210.21	0.00	17.59	10.00	0.21
2019	**Dry**	BANDIM	Bissau	Tap	7.28	28.26	0.25	0.74	539.78	71.00	5.00	255.89	0.01	0.82	0.10	0.15	3.00	0.41	210.00	0.00	16.97	10.00	0.21
2019	**Dry**	BANDIM	Bissau	Fountain	7.80	30.00	0.23	0.87	470.00	101.90	2.30	237.00	0.05	0.97	0.11	0.05	0.00	1.35	200.00	0.00	18.00	5.00	0.13
2019	**Dry**	BANDIM	Bissau	Fountain	7.81	28.98	0.21	0.90	459.00	98.50	2.26	235.00	0.05	1.00	0.10	0.05	0.00	1.30	199.00	0.00	17.00	5.00	0.13
2019	**Dry**	BANDIM	Bissau	Fountain	7.80	30.00	0.23	0.89	471.00	100.00	2.32	238.00	0.05	1.00	0.11	0.06	0.00	1.36	201.00	0.00	18.24	5.00	0.13
2019	**Dry**	BANDIM	Bissau	Fountain	7.81	29.95	0.22	0.88	472.21	101.20	2.30	238.23	0.05	1.00	0.11	0.06	0.00	1.36	201.33	0.00	17.99	6.00	0.14
2019	**Dry**	BANDIM	Bissau	Fountain	7.81	30.40	0.23	0.87	469.29	99.78	2.43	237.12	0.05	1.00	0.10	0.06	0.00	1.30	201.56	0.00	18.25	5.00	0.13
2019	**Dry**	BANDIM	Bissau	Fountain	7.80	30.88	0.23	0.87	469.98	102.32	2.30	238.68	0.05	1.00	0.10	0.05	0.00	1.32	199.79	0.00	18.37	6.00	0.14
2019	**Dry**	BANDIM	Bissau	Hole	7.92	24.59	0.29	1.00	609.00	94.20	4.10	304.00	0.01	0.77	0.12	0.05	1.00	1.10	215.00	0.00	11.00	10.00	0.10
2019	**Dry**	BANDIM	Bissau	Hole	7.80	25.00	0.30	0.99	607.00	98.00	4.30	300.10	0.01	0.78	0.12	0.05	1.00	0.99	210.00	0.00	10.89	11.00	0.10
2019	**Dry**	BANDIM	Bissau	Hole	7.85	24.45	0.31	1.25	605.79	97.37	4.27	299.00	0.01	0.78	0.12	0.05	0.99	1.20	216.23	0.00	10.00	10.89	0.10
2019	**Dry**	BANDIM	Bissau	Hole	7.82	25.69	0.31	1.32	606.98	99.75	4.23	301.29	0.01	0.78	0.12	0.05	1.00	1.19	216.87	0.00	10.98	10.78	0.10
2019	**Dry**	BANDIM	Bissau	Hole	7.83	24.20	0.30	1.00	609.10	100.69	4.39	305.12	0.01	0.77	0.12	0.05	0.99	1.00	216.51	0.00	10.00	10.20	0.10
2019	**Dry**	BANDIM	Bissau	Hole	7.82	24.85	0.29	1.00	610.00	100.49	4.37	305.88	0.01	0.76	0.12	0.05	1.00	0.99	216.00	0.00	10.58	10.78	0.10
2019	**Dry**	BANDIM	Bissau	Reservoir outlet	7.81	29.91	0.26	0.86	530.00	103.90	4.60	268.00	0.02	2.00	0.13	0.10	0.00	0.40	208.00	0.00	15.00	10.00	0.22
2019	**Dry**	BANDIM	Bissau	Reservoir outlet	7.75	30.00	0.26	0.90	528.00	99.40	4.70	265.00	0.01	1.99	0.12	0.10	0.00	0.39	209.00	0.00	16.25	10.00	0.22
2019	**Dry**	BANDIM	Bissau	Reservoir outlet	7.75	29.25	0.26	0.89	530.12	100.20	4.70	269.25	0.02	2.00	0.12	0.10	0.00	0.40	210.20	0.00	16.45	10.25	0.25
2019	**Dry**	BANDIM	Bissau	Reservoir outlet	7.74	29.92	0.26	0.89	531.25	101.58	4.67	269.54	0.02	1.98	0.12	0.10	0.00	0.40	210.00	0.00	16.58	10.80	0.25
2019	**Dry**	BANDIM	Bissau	Reservoir outlet	7.74	30.00	0.26	0.87	529.89	99.88	4.72	268.48	0.02	2.01	0.13	0.10	0.00	0.39	209.19	0.00	16.79	9.99	0.22
2019	**Dry**	BANDIM	Bissau	Reservoir outlet	7.75	29.78	0.26	0.88	528.98	100.25	4.73	268.98	0.02	2.02	0.13	0.10	0.00	0.39	209.99	0.00	16.90	9.98	0.22
2019	**Dry**	HSM	Bissau	Fountain	7.63	31.14	0.24	0.69	508.00	117.00	5.00	260.00	0.00	1.00	0.09	0.10	2.00	0.99	200.00	0.00	15.00	15.00	0.29
2019	**Dry**	HSM	Bissau	Fountain	7.61	30.10	0.25	0.70	507.20	116.90	5.10	261.00	0.00	0.99	0.08	0.09	1.85	1.00	199.25	0.00	15.30	14.98	0.31

2019	**Dry**	HSM	Bissau	Fountain	7.62	30.98	0.25	0.70	510.26	117.58	5.98	260.89	0.00	1.02	0.10	0.09	1.99	1.00	201.24	0.00	15.45	15.00	0.30
2019	**Dry**	HSM	Bissau	Fountain	7.62	30.59	0.25	0.69	509.98	117.89	5.80	260.94	0.00	1.00	0.10	0.10	2.10	0.99	201.45	0.00	15.47	15.21	0.30
2019	**Dry**	HSM	Bissau	Fountain	7.63	31.25	0.25	0.69	508.98	116.89	5.12	261.29	0.00	1.00	0.09	0.10	2.00	1.00	200.14	0.00	15.37	15.42	0.29
2019	**Dry**	HSM	Bissau	Fountain	7.63	31.45	0.25	0.71	510.24	117.59	5.48	261.89	0.00	0.95	0.90	0.09	2.00	1.00	200.12	0.00	15.40	15.26	0.30
2019	**Dry**	HSM	Bissau	Reservoir outlet	7.72	30.20	0.25	0.59	523.00	117.50	5.22	261.00	0.03	0.91	0.10	0.20	8.00	0.72	210.00	0.24	11.00	20.00	0.87
2019	**Dry**	HSM	Bissau	Reservoir outlet	7.70	30.10	0.25	0.57	522.00	115.90	5.32	263.10	0.03	0.94	0.10	0.21	7.90	0.73	209.14	0.23	11.00	20.20	0.89
2019	**Dry**	HSM	Bissau	Reservoir outlet	7.69	29.98	0.25	0.58	521.58	116.89	5.28	262.90	0.03	0.95	0.10	0.20	8.01	0.72	210.20	0.22	11.00	19.99	0.88
2019	**Dry**	HSM	Bissau	Reservoir outlet	7.59	30.20	0.25	0.58	520.99	116.99	5.29	263.00	0.03	0.95	0.10	0.22	7.89	0.72	209.99	0.22	11.00	19.78	0.88
2019	**Dry**	HSM	Bissau	Reservoir outlet	7.70	30.10	0.25	0.59	521.56	117.99	5.28	262.99	0.03	0.94	0.10	0.21	8.02	0.71	210.49	0.21	11.00	20.10	0.86
2019	**Dry**	HSM	Bissau	Reservoir outlet	7.72	29.98	0.25	0.59	521.26	117.85	5.29	262.80	0.03	0.95	0.10	0.21	8.02	0.71	210.79	0.22	11.00	20.08	0.85
2019	**Dry**	QJ	Bissau	Tap	7.56	32.16	0.22	2.00	466.00	135.10	6.14	235.00	0.01	0.86	0.12	0.25	0.00	1.25	195.00	0.00	9.90	20.00	0.05
2019	**Dry**	QJ	Bissau	Tap	7.61	31.98	0.22	2.00	462.00	133.78	5.91	233.98	0.01	0.85	0.13	0.25	0.00	1.23	193.50	0.01	10.00	19.80	0.04
2019	**Dry**	QJ	Bissau	Tap	7.60	32.10	0.22	1.99	462.90	134.95	6.18	236.46	0.01	0.87	0.14	0.25	0.00	1.20	193.50	0.01	10.00	20.00	0.04
2019	**Dry**	QJ	Bissau	Tap	7.58	31.99	0.22	2.01	463.88	135.80	6.49	236.49	0.01	0.86	0.13	0.25	0.00	1.25	194.79	0.01	10.00	20.00	0.04
2019	**Dry**	QJ	Bissau	Tap	7.56	32.00	0.22	2.00	462.99	134.90	6.90	235.50	0.01	0.87	0.14	0.25	0.00	1.24	194.88	0.01	9.88	20.00	0.04
2019	**Dry**	QJ	Bissau	Tap	7.58	31.99	0.22	2.00	463.05	134.60	6.97	235.95	0.01	0.86	0.14	0.25	0.00	1.24	195.49	0.01	10.23	20.78	0.04
2019	**Wet**	H3A	Bissau	Hole	8.18	31.38	0.24	1.69	517.00	36.50	0.00	255.00	0.52	0.00	0.00	1.00	0.60	0.60	225.00	0.02	12.00	5.00	0.11
2019	**Wet**	H3A	Bissau	Hole	8.12	29.38	0.22	1.65	479.00	41.00	0.00	256.78	0.51	0.00	0.00	1.00	0.62	0.60	224.00	0.02	11.99	4.89	0.11
2019	**Wet**	H3A	Bissau	Hole	8.15	30.12	0.24	1.65	518.00	38.19	0.00	257.79	0.50	0.00	0.00	1.00	0.61	0.59	223.90	0.02	12.07	5.07	0.11
2019	**Wet**	H3A	Bissau	Hole	8.18	31.00	0.23	1.62	512.00	37.89	0.00	257.36	0.52	0.00	0.00	1.00	0.61	0.61	223.89	0.02	11.98	5.04	0.11
2019	**Wet**	H3A	Bissau	Hole	8.17	29.79	0.22	1.60	516.00	37.98	0.00	256.18	0.52	0.00	0.00	1.00	0.60	0.61	224.98	0.02	12.36	5.02	0.11
2019	**Wet**	H3A	Bissau	Hole	8.17	31.20	0.22	1.62	518.00	38.12	0.00	256.95	0.53	0.00	0.00	1.00	0.60	0.61	225.12	0.02	12.08	4.98	0.11
2019	**Wet**	H3A	Bissau	Tap	8.28	29.00	0.25	1.59	524.00	8.90	0.00	262.00	0.60	0.00	0.00	0.00	0.68	0.66	225.00	0.00	14.00	5.00	0.14
2019	**Wet**	H3A	Bissau	Tap	8.22	30.10	0.26	1.49	523.48	9.15	0.00	259.40	0.61	0.00	0.00	0.00	0.69	0.67	221.60	0.01	13.90	5.00	0.14
2019	**Wet**	H3A	Bissau	Tap	7.99	30.49	0.25	1.59	524.19	8.79	0.00	258.99	0.62	0.00	0.00	0.00	0.70	0.66	226.15	0.00	13.99	4.98	0.14
2019	**Wet**	H3A	Bissau	Tap	8.27	29.04	0.25	1.58	523.90	8.76	0.00	260.48	0.61	0.00	0.00	0.00	0.68	0.67	225.98	0.01	14.13	5.02	0.14
2019	**Wet**	H3A	Bissau	Tap	8.23	28.80	0.25	1.56	523.59	8.98	0.00	260.58	0.62	0.00	0.00	0.00	0.68	0.66	224.78	0.00	14.32	5.00	0.14
2019	**Wet**	H3A	Bissau	Tap	8.26	29.00	0.25	1.57	524.02	9.02	0.00	258.98	0.61	0.00	0.00	0.00	0.66	0.66	224.50	0.01	13.89	4.99	0.14
2019	**Wet**	H3A	Bissau	Fountain	8.20	30.00	0.24	2.00	520.00	27.26	0.00	284.00	0.60	0.00	0.00	0.00	0.59	0.59	231.00	0.05	17.99	6.00	0.13
2019	**Wet**	H3A	Bissau	Fountain	8.10	31.00	0.23	1.98	498.90	27.00	0.00	282.89	0.57	0.00	0.00	0.00	0.59	0.59	229.96	0.05	18.00	6.02	0.13
2019	**Wet**	H3A	Bissau	Fountain	8.20	30.12	0.24	2.00	519.89	27.46	0.00	283.50	0.60	0.00	0.00	0.00	0.61	0.58	230.80	0.05	17.48	5.90	0.13
2019	**Wet**	H3A	Bissau	Fountain	8.13	30.00	0.24	2.02	520.79	27.12	0.00	284.26	0.59	0.00	0.00	0.00	0.59	0.60	231.50	0.04	17.99	6.02	0.13
2019	**Wet**	H3A	Bissau	Fountain	8.21	29.99	0.24	2.04	520.20	27.20	0.00	284.25	0.60	0.00	0.00	0.00	0.60	0.59	231.56	0.04	18.02	6.01	0.13

2019	**Wet**	H3A	Bissau	Fountain	8.25	30.47	0.24	1.98	520.16	27.00	0.00	284.02	0.60	0.00	0.00	0.00	0.60	0.58	231.46	0.05	17.98	6.00	0.13
2019	**Wet**	HSE	Bissau	Hole	8.23	32.32	0.26	1.18	545.00	57.70	0.00	272.12	0.61	0.01	0.00	0.00	0.38	0.38	240.75	0.06	19.00	5.00	0.16
2019	**Wet**	HSE	Bissau	Hole	8.37	31.20	0.27	1.21	547.78	56.90	0.00	271.28	0.60	0.01	0.00	0.00	0.40	0.38	238.29	0.06	20.20	5.10	0.16
2019	**Wet**	HSE	Bissau	Hole	8.20	32.19	0.26	1.20	546.28	57.48	0.00	272.48	0.60	0.01	0.00	0.00	0.39	0.38	239.78	0.06	18.98	5.29	0.16
2019	**Wet**	HSE	Bissau	Hole	8.29	31.89	0.26	1.21	546.99	57.90	0.00	272.50	0.61	0.01	0.00	0.00	0.39	0.39	240.79	0.06	19.23	5.45	0.16
2019	**Wet**	HSE	Bissau	Hole	8.23	32.23	0.26	1.18	544.90	60.10	0.00	271.90	0.61	0.01	0.00	0.00	0.37	0.39	240.75	0.06	19.00	4.98	0.16
2019	**Wet**	HSE	Bissau	Hole	8.23	32.32	0.26	1.18	544.97	57.70	0.00	272.15	0.62	0.01	0.00	0.00	0.38	0.38	240.15	0.06	19.00	5.00	0.16
2019	**Wet**	HSE	Bissau	Reservoir outlet	8.24	31.68	0.26	1.20	547.00	1.20	0.00	274.00	0.70	0.00	0.00	0.00	0.78	0.78	245.00	0.06	17.00	0.00	0.15
2019	**Wet**	HSE	Bissau	Reservoir outlet	8.21	30.30	0.25	1.22	548.30	1.18	0.00	271.00	0.69	0.00	0.00	0.00	0.77	0.78	244.89	0.06	17.86	0.00	0.15
2019	**Wet**	HSE	Bissau	Reservoir outlet	8.23	30.15	0.26	1.20	546.99	1.20	0.00	273.48	0.71	0.00	0.00	0.00	0.79	0.77	245.23	0.06	16.79	0.00	0.15
2019	**Wet**	HSE	Bissau	Reservoir outlet	8.23	31.20	0.26	1.20	548.76	1.20	0.00	274.56	0.69	0.00	0.00	0.00	0.78	0.78	246.02	0.06	16.77	0.00	0.15
2019	**Wet**	HSE	Bissau	Reservoir outlet	8.25	31.45	0.26	1.21	547.90	1.19	0.00	274.32	0.71	0.00	0.00	0.00	0.78	0.77	244.96	0.06	17.09	0.00	0.15
2019	**Wet**	HSE	Bissau	Reservoir outlet	8.25	31.52	0.26	1.21	548.00	1.19	0.00	274.15	0.70	0.00	0.00	0.00	0.79	0.78	244.85	0.06	17.04	0.00	0.15
2019	**Wet**	HSE	Bissau	Fountain	8.35	29.77	0.26	1.79	543.00	16.50	0.00	272.00	0.71	0.01	0.02	0.00	0.65	0.65	240.00	0.04	20.00	10.00	0.16
2019	**Wet**	HSE	Bissau	Fountain	8.21	30.00	0.26	1.75	539.58	15.73	0.00	271.58	0.72	0.01	0.02	0.00	0.61	0.65	238.13	0.04	21.21	10.00	0.16
2019	**Wet**	HSE	Bissau	Fountain	8.34	29.79	0.26	1.74	543.18	16.52	0.00	271.56	0.71	0.01	0.02	0.00	0.65	0.64	241.23	0.04	19.98	10.00	0.16
2019	**Wet**	HSE	Bissau	Fountain	8.35	29.69	0.26	1.73	542.90	16.28	0.00	271.78	0.72	0.01	0.02	0.00	0.65	0.65	241.32	0.04	20.29	10.00	0.16
2019	**Wet**	HSE	Bissau	Fountain	8.35	30.00	0.26	1.75	543.90	16.75	0.00	272.06	0.72	0.01	0.02	0.00	0.64	0.64	239.90	0.04	20.03	10.00	0.16
2019	**Wet**	HSE	Bissau	Fountain	8.35	30.00	0.26	1.78	543.50	16.40	0.00	272.10	0.71	0.01	0.02	0.00	0.65	0.65	240.12	0.04	20.49	10.00	0.16
2019	**Wet**	BLELA7	Quinhamel	Shallow wells	5.66	27.84	0.07	20.00	153.00	78.30	2.20	77.95	5.00	0.15	0.59	4.00	0.20	0.20	35.00	0.19	16.25	17.80	0.16
2019	**Wet**	BLELA7	Quinhamel	Shallow wells	5.56	26.83	0.07	21.00	152.80	67.17	2.20	78.20	4.90	0.16	0.58	3.90	0.20	0.21	34.25	0.19	15.30	18.20	0.16
2019	**Wet**	BLELA7	Quinhamel	Shallow wells	5.46	27.14	0.07	19.89	153.50	54.20	1.98	77.20	5.02	0.15	0.59	3.99	0.20	0.20	35.02	0.17	16.02	17.98	0.16
2019	**Wet**	BLELA7	Quinhamel	Shallow wells	5.55	27.36	0.07	20.02	152.90	71.12	2.10	77.97	4.99	0.15	0.58	4.02	0.20	0.20	35.04	0.19	16.04	17.69	0.15
2019	**Wet**	BLELA7	Quinhamel	Shallow wells	5.68	27.81	0.07	18.56	153.03	69.10	2.19	78.10	4.98	0.15	0.59	4.08	0.20	0.19	35.10	0.18	16.28	17.65	0.15
2019	**Wet**	BLELA7	Quinhamel	Shallow wells	5.67	27.83	0.07	20.00	153.48	70.00	2.20	78.39	5.23	0.16	0.59	4.00	0.20	0.20	35.08	0.19	16.35	17.62	0.15
2019	**Wet**	INTOZINHO8	Quinhamel	Shallow wells	4.94	28.22	0.03	18.59	66.00	54.78	1.80	33.00	5.32	0.08	0.35	5.00	0.20	0.20	31.90	0.04	15.60	22.00	0.14
2019	**Wet**	INTOZINHO8	Quinhamel	Shallow wells	3.98	27.98	0.03	20.14	65.63	56.00	2.02	35.00	5.46	0.08	0.31	4.95	0.20	0.20	33.10	0.04	14.20	23.20	0.14
2019	**Wet**	INTOZINHO8	Quinhamel	Shallow wells	4.95	28.03	0.03	20.19	67.32	55.14	2.00	33.58	5.36	0.08	0.34	5.04	0.20	0.19	32.52	0.04	15.36	22.86	0.14
2019	**Wet**	INTOZINHO8	Quinhamel	Shallow wells	4.93	28.08	0.03	19.45	66.89	51.01	1.89	33.05	5.29	0.08	0.34	5.02	0.20	0.19	31.48	0.04	15.42	22.79	0.14
2019	**Wet**	INTOZINHO8	Quinhamel	Shallow wells	4.94	28.34	0.03	18.98	66.20	53.03	1.86	33.42	5.31	0.08	0.35	5.00	0.20	0.20	31.89	0.04	15.56	22.86	0.14
2019	**Wet**	INTOZINHO8	Quinhamel	Shallow wells	4.56	28.65	0.03	20.89	66.32	50.14	1.80	33.01	5.32	0.08	0.35	5.10	0.20	0.20	31.78	0.04	15.70	22.76	0.14

2019	**Wet**	BOR9	Quinhamel	Shallow wells	4.66	27.66	0.07	21.02	142.00	45.00	0.20	71.00	6.20	0.12	0.28	2.98	0.19	0.19	19.12	0.04	15.00	15.95	0.16
2019	**Wet**	BOR9	Quinhamel	Shallow wells	4.59	27.48	0.07	22.14	141.00	31.08	0.22	70.88	6.17	0.12	0.29	2.89	0.19	0.19	19.02	0.04	14.96	16.20	0.16
2019	**Wet**	BOR9	Quinhamel	Shallow wells	4.65	27.78	0.07	12.45	142.35	29.16	0.21	70.49	6.19	0.12	0.27	2.78	0.19	0.18	18.79	0.04	15.23	15.96	0.16
2019	**Wet**	BOR9	Quinhamel	Shallow wells	4.65	27.69	0.07	21.75	142.58	20.81	0.21	71.58	6.21	0.12	0.27	2.90	0.19	0.19	19.20	0.04	15.08	16.00	0.16
2019	**Wet**	BOR9	Quinhamel	Shallow wells	4.63	27.85	0.07	20.85	141.89	21.04	0.20	71.48	6.18	0.12	0.28	2.95	0.19	0.19	19.88	0.04	15.21	15.45	0.16
2019	**Wet**	BOR9	Quinhamel	Shallow wells	4.67	27.68	0.07	19.89	141.78	19.34	0.20	70.69	6.21	0.12	0.28	2.97	0.19	0.20	18.79	0.04	15.19	15.75	0.16
2019	**Wet**	BOR10	Quinhamel	Shallow wells	4.53	27.88	0.06	20.58	138.00	24.01	0.00	69.00	2.10	0.19	0.26	2.13	0.13	0.13	25.98	0.09	14.32	17.76	0.13
2019	**Wet**	BOR10	Quinhamel	Shallow wells	4.49	28.00	0.06	19.79	137.49	23.12	0.00	68.26	1.97	0.19	0.26	2.09	0.13	0.14	26.03	0.08	14.21	17.89	0.13
2019	**Wet**	BOR10	Quinhamel	Shallow wells	4.50	27.89	0.06	21.48	137.99	19.02	0.00	69.15	2.05	0.19	0.26	2.12	0.13	0.13	26.00	0.08	14.16	16.97	0.13
2019	**Wet**	BOR10	Quinhamel	Shallow wells	4.52	27.00	0.06	20.18	138.24	19.40	0.00	68.79	2.12	0.19	0.26	2.12	0.13	0.13	25.97	0.08	14.30	16.79	0.13
2019	**Wet**	BOR10	Quinhamel	Shallow wells	4.54	27.56	0.06	20.17	138.00	18.99	0.00	69.28	2.10	0.19	0.26	2.13	0.13	0.13	25.78	0.09	14.26	17.98	0.13
2019	**Wet**	BOR10	Quinhamel	Shallow wells	4.54	27.79	0.06	21.02	138.49	18.36	0.00	69.48	2.12	0.19	0.26	2.13	0.13	0.14	25.69	0.09	14.23	17.69	0.13
2019	**Wet**	QUINHAMEL 11	Quinhamel	Tubewell	5.59	28.40	0.05	2.98	120.00	84.50	0.00	60.00	10.10	0.04	0.00	1.59	0.20	0.20	15.30	0.01	16.95	25.00	0.12
2019	**Wet**	QUINHAMEL 11	Quinhamel	Tubewell	5.10	27.49	0.05	3.00	118.89	85.61	0.00	59.95	9.98	0.04	0.00	1.69	0.20	0.20	16.20	0.01	17.00	25.00	0.12
2019	**Wet**	QUINHAMEL 11	Quinhamel	Tubewell	5.60	28.47	0.05	3.10	120.18	85.00	0.00	61.02	10.20	0.04	0.00	1.55	0.20	0.19	15.90	0.01	17.00	25.00	0.12
2019	**Wet**	QUINHAMEL 11	Quinhamel	Tubewell	5.59	28.32	0.05	2.99	119.78	84.12	0.00	60.15	10.14	0.04	0.00	1.56	0.20	0.20	15.45	0.01	17.02	25.00	0.12
2019	**Wet**	QUINHAMEL 11	Quinhamel	Tubewell	5.53	28.25	0.05	2.89	120.79	84.38	0.00	60.19	10.11	0.04	0.00	1.60	0.20	0.21	15.32	0.01	17.42	25.00	0.12
2019	**Wet**	QUINHAMEL 11	Quinhamel	Tubewell	5.67	28.42	0.05	3.00	120.78	84.25	0.00	59.48	10.16	0.04	0.00	1.58	0.20	0.20	15.33	0.01	16.79	25.00	0.12
2019	**Wet**	QUINHAMEL 12	Quinhamel	Tubewell	5.27	28.42	0.21	2.52	446.00	70.10	0.00	223.00	0.78	0.04	0.00	0.00	0.16	0.16	6.00	0.00	12.00	75.00	0.10
2019	**Wet**	QUINHAMEL 12	Quinhamel	Tubewell	5.25	27.29	0.21	2.51	443.00	67.16	0.00	221.15	0.78	0.04	0.01	0.00	0.16	0.16	5.89	0.00	11.78	75.00	0.10
2019	**Wet**	QUINHAMEL 12	Quinhamel	Tubewell	5.26	28.32	0.21	2.49	448.25	54.32	0.00	224.25	0.78	0.04	0.01	0.00	0.16	0.16	5.79	0.00	12.00	75.00	0.10
2019	**Wet**	QUINHAMEL 12	Quinhamel	Tubewell	5.27	28.29	0.21	2.53	447.89	31.25	0.00	224.12	0.78	0.04	0.01	0.00	0.16	0.16	6.02	0.00	12.40	74.00	0.10
2019	**Wet**	QUINHAMEL 12	Quinhamel	Tubewell	5.27	28.40	0.21	2.31	448.59	29.89	0.00	223.78	0.78	0.04	0.01	0.00	0.16	0.17	6.09	0.00	12.03	74.89	0.10
2019	**Wet**	QUINHAMEL 12	Quinhamel	Tubewell	5.26	28.36	0.21	2.38	447.87	35.12	0.00	223.98	0.78	0.04	0.01	0.00	0.16	0.17	6.15	0.00	12.45	75.00	0.10
2019	**Wet**	QUINHAMEL 13	Quinhamel	Tubewell	4.81	27.68	0.02	1.98	46.00	30.10	0.00	23.00	1.00	0.08	0.00	2.10	0.19	0.19	2.00	0.00	18.00	20.00	0.09
2019	**Wet**	QUINHAMEL 13	Quinhamel	Tubewell	4.83	28.20	0.02	2.10	47.58	29.47	0.00	24.50	1.10	0.08	0.00	2.00	0.20	0.19	2.20	0.01	18.96	20.00	0.09
2019	**Wet**	QUINHAMEL 13	Quinhamel	Tubewell	4.86	27.66	0.02	2.00	48.23	28.99	0.00	23.47	1.00	0.08	0.00	2.20	0.19	0.19	2.10	0.00	17.90	20.00	0.09
2019	**Wet**	QUINHAMEL 13	Quinhamel	Tubewell	4.79	27.89	0.02	1.99	46.10	32.01	0.00	23.98	0.98	0.08	0.00	2.00	0.19	0.18	1.98	0.00	18.20	20.00	0.09
2019	**Wet**	QUINHAMEL 13	Quinhamel	Tubewell	4.80	27.68	0.02	1.95	47.58	28.79	0.00	23.49	1.00	0.08	0.00	1.96	0.19	0.18	2.00	0.00	18.00	20.00	0.09
2019	**Wet**	QUINHAMEL 13	Quinhamel	Tubewell	4.80	27.59	0.02	1.96	47.97	29.00	0.00	23.89	1.00	0.08	0.00	2.20	0.20	0.19	1.89	0.00	18.02	20.00	0.09

2019	**Wet**	BAGDAD	Quinhamel	Tubewell	7.79	28.26	0.04	1.20	97.00	28.32	0.00	48.00	1.09	0.02	0.00	2.13	0.26	0.26	12.00	0.06	12.00	10.00	0.02
2019	**Wet**	BAGDAD	Quinhamel	Tubewell	6.97	28.10	0.04	0.98	96.00	27.16	0.00	46.95	1.07	0.02	0.00	2.15	0.26	0.25	12.00	0.06	11.70	10.00	0.02
2019	**Wet**	BAGDAD	Quinhamel	Tubewell	7.76	28.00	0.04	1.00	97.59	27.79	0.00	47.89	1.09	0.02	0.00	2.13	0.26	0.25	12.00	0.06	12.02	10.00	0.02
2019	**Wet**	BAGDAD	Quinhamel	Tubewell	7.78	28.06	0.04	1.12	97.49	26.98	0.00	48.20	1.09	0.02	0.00	2.14	0.26	0.26	12.00	0.06	12.20	10.00	0.02
2019	**Wet**	BAGDAD	Quinhamel	Tubewell	7.77	28.09	0.04	1.00	96.80	27.24	0.00	48.76	1.08	0.02	0.00	2.13	0.26	0.26	12.00	0.06	11.80	10.00	0.02
2019	**Wet**	BAGDAD	Quinhamel	Tubewell	7.79	28.28	0.04	1.00	97.89	28.12	0.00	48.88	1.09	0.02	0.00	2..09	0.26	0.26	12.00	0.06	12.00	10.00	0.02
2019	**Wet**	QUINHAMEL 15	Quinhamel	Tubewell	4.65	28.08	0.02	1.23	50.00	26.17	0.00	26.00	1.20	0.04	0.06	3.21	0.25	0.25	7.00	0.00	17.00	10.00	0.06
2019	**Wet**	QUINHAMEL 15	Quinhamel	Tubewell	4.64	27.89	0.02	1.25	49.23	26.78	0.00	27.00	1.20	0.04	0.06	3.32	0.25	0.25	7.00	0.00	16.85	10.00	0.06
2019	**Wet**	QUINHAMEL 15	Quinhamel	Tubewell	4.78	28.00	0.02	1.24	51.02	25.78	0.00	26.00	1.20	0.04	0.06	3.19	0.25	0.26	7.00	0.00	17.21	10.00	0.06
2019	**Wet**	QUINHAMEL 15	Quinhamel	Tubewell	4.80	28.05	0.02	1.25	50.23	26.12	0.00	26.23	1.20	0.04	0.06	3.22	0.25	0.25	7.00	0.00	17.08	10.00	0.06
2019	**Wet**	QUINHAMEL 15	Quinhamel	Tubewell	4.71	27.98	0.02	1.24	50.00	25.95	0.00	26.49	1.20	0.04	0.06	3.24	0.25	0.26	7.00	0.00	17.00	10.00	0.06
2019	**Wet**	QUINHAMEL 15	Quinhamel	Tubewell	4.70	27.89	0.02	1.25	50.89	25.47	0.00	27.19	1.20	0.04	0.06	3.23	0.25	0.25	7.00	0.00	17.00	10.00	0.06
2019	**Wet**	BANDIM	Bissau	Tap	8.27	28.00	0.26	1.30	530.00	42.40	0.00	265.00	1.00	0.00	0.04	8.00	0.55	0.55	225.00	0.12	19.00	15.00	0.11
2019	**Wet**	BANDIM	Bissau	Tap	8.17	28.07	0.26	1.33	528.05	40.74	0.00	263.21	1.00	0.00	0.04	8.00	0.55	0.55	224.20	0.12	19.00	15.00	0.11
2019	**Wet**	BANDIM	Bissau	Tap	8.26	28.02	0.26	1.29	530.41	42.00	0.00	267.58	1.00	0.00	0.04	7.98	0.55	0.56	224.00	0.12	19.00	15.00	0.11
2019	**Wet**	BANDIM	Bissau	Tap	8.24	28.06	0.26	1.30	530.00	41.89	0.00	267.21	1.00	0.00	0.04	8.00	0.55	0.56	224.98	0.12	19.00	15.00	0.11
2019	**Wet**	BANDIM	Bissau	Tap	8.25	28.00	0.26	1.30	529.36	42.00	0.00	264.79	1.00	0.00	0.04	7.96	0.55	0.55	225.00	0.12	19.00	15.00	0.11
2019	**Wet**	BANDIM	Bissau	Tap	8.26	28.00	0.26	1.29	530.02	42.63	0.00	264.69	1.00	0.00	0.04	8.00	0.55	0.54	225.10	0.12	19.00	15.00	0.11
2019	**Wet**	BANDIM	Bissau	Fountain	8.46	30.72	0.22	1.49	468.00	-2.60	0.00	234.00	0.57	0.01	0.01	5.00	0.34	0.34	210.00	0.10	20.00	15.00	0.14
2019	**Wet**	BANDIM	Bissau	Fountain	8.30	30.65	0.22	1.46	465.80	-2.49	0.00	230.51	0.58	0.01	0.01	5.00	0.34	0.34	199.46	0.10	19.87	15.00	0.14
2019	**Wet**	BANDIM	Bissau	Fountain	8.45	30.71	0.22	1.50	467.00	-2.39	0.00	232.21	0.59	0.01	0.01	5.00	0.36	0.33	212.00	0.10	20.20	15.00	0.14
2019	**Wet**	BANDIM	Bissau	Fountain	8.42	30.75	0.22	1.51	468.20	-2.62	0.00	233.69	0.60	0.01	0.01	5.00	0.34	0.34	212.11	0.10	19.79	15.00	0.14
2019	**Wet**	BANDIM	Bissau	Fountain	8.46	30.75	0.22	1.39	468.36	-2.62	0.00	234.15	0.57	0.01	0.01	5.00	0.35	0.34	209.00	0.10	20.36	15.00	0.14
2019	**Wet**	BANDIM	Bissau	Fountain	8.46	30.77	0.22	1.41	467.89	-2.62	0.00	235.21	0.57	0.01	0.01	5.00	0.34	0.33	212.89	0.10	20.32	15.00	0.14
2019	**Wet**	BANDIM	Bissau	Hole	8.14	32.14	0.26	1.00	541.00	-4.00	0.00	271.00	0.61	0.01	0.01	3.00	0.31	0.31	220.00	0.10	24.00	20.00	0.23
2019	**Wet**	BANDIM	Bissau	Hole	8.09	31.87	0.26	1.02	539.48	-3.56	0.00	270.10	0.63	0.01	0.01	3.00	0.33	0.30	218.79	0.10	23.54	20.00	0.23
2019	**Wet**	BANDIM	Bissau	Hole	8.10	32.18	0.26	0.99	540.89	-4.10	0.00	271.89	0.61	0.01	0.01	3.00	0.32	0.31	219.03	0.10	24.04	20.00	0.23
2019	**Wet**	BANDIM	Bissau	Hole	8.11	32.20	0.26	1.00	541.06	-3.98	0.00	271.96	0.62	0.01	0.01	3.00	0.32	0.32	220.03	0.10	24.52	20.00	0.23
2019	**Wet**	BANDIM	Bissau	Hole	8.13	31.98	0.26	1.03	542.89	-4.27	0.00	272.48	0.60	0.01	0.01	3.00	0.31	0.31	220.00	0.10	24.32	20.00	0.23
2019	**Wet**	BANDIM	Bissau	Hole	8.14	32.17	0.26	0.99	542.08	-4.29	0.00	272.89	0.61	0.01	0.01	3.00	0.31	0.30	219.80	0.10	24.74	20.00	0.23
2019	**Wet**	BANDIM	Bissau	Reservoir outlet	8.18	27.92	0.25	1.69	520.00	11.80	0.00	260.00	0.89	0.00	0.03	7.00	0.30	0.30	215.00	0.12	19.00	20.00	0.07
2019	**Wet**	BANDIM	Bissau	Reservoir outlet	7.98	27.10	0.25	1.79	518.00	11.02	0.00	271.00	0.90	0.00	0.02	7.00	0.30	0.31	219.01	0.12	20.00	19.00	0.07

2019	**Wet**	BANDIM	Bissau	Reservoir outlet	8.15	27.58	0.25	1.59	521.00	12.00	0.00	269.68	0.90	0.00	0.02	7.00	0.30	0.31	218.21	0.12	19.25	20.00	0.07
2019	**Wet**	BANDIM	Bissau	Reservoir outlet	8.18	27.89	0.25	1.68	520.57	11.28	0.00	270.18	0.88	0.00	0.02	7.00	0.30	0.31	216.45	0.12	18.98	18.89	0.07
2019	**Wet**	BANDIM	Bissau	Reservoir outlet	8.17	28.00	0.25	1.71	519.88	11.89	0.00	261.48	0.89	0.00	0.02	7.00	0.30	0.30	215.78	0.12	19.00	20.00	0.07
2019	**Wet**	BANDIM	Bissau	Reservoir outlet	8.17	27.89	0.25	1.68	520.00	12.03	0.00	260.79	0.90	0.00	0.02	7.00	0.30	0.29	215.37	0.12	18.80	20.00	0.07
2019	**Wet**	HSM	Bissau	Fountain	8.10	31.41	0.24	1.02	578.00	35.00	0.00	257.00	1.00	0.01	0.02	7.00	0.35	0.35	215.00	0.10	17.00	40.00	0.07
2019	**Wet**	HSM	Bissau	Fountain	8.20	30.30	0.24	1.09	575.12	34.48	0.00	257.01	1.00	0.01	0.02	7.00	0.35	0.35	216.32	0.10	17.00	38.93	0.07
2019	**Wet**	HSM	Bissau	Fountain	8.12	31.25	0.24	1.06	577.58	35.21	0.00	256.21	1.00	0.01	0.02	7.00	0.35	0.34	215.85	0.10	17.00	40.12	0.07
2019	**Wet**	HSM	Bissau	Fountain	8.18	31.45	0.24	1.10	578.49	34.80	0.00	258.03	1.00	0.01	0.02	7.00	0.35	0.34	215.79	0.10	17.00	39.46	0.07
2019	**Wet**	HSM	Bissau	Fountain	8.14	31.25	0.24	1.09	578.00	35.65	0.00	257.04	1.00	0.01	0.02	7.00	0.35	0.35	216.00	0.10	17.00	40.00	0.07
2019	**Wet**	HSM	Bissau	Fountain	8.15	31.29	0.24	1.08	577.98	35.23	0.00	257.05	1.00	0.01	0.02	7.00	0.35	0.34	216.32	0.10	17.00	40.12	0.07
2019	**Wet**	HSM	Bissau	Reservoir outlet	8.14	29.62	0.25	1.00	515.00	42.10	0.00	257.00	1.00	0.00	0.01	6.00	0.29	0.29	185.00	0.08	18.00	30.00	0.10
2019	**Wet**	HSM	Bissau	Reservoir outlet	7.94	30.00	0.25	1.00	511.60	43.10	0.00	260.00	1.00	0.00	0.01	6.52	0.29	0.28	183.23	0.08	18.24	30.12	0.10
2019	**Wet**	HSM	Bissau	Reservoir outlet	8.10	29.67	0.25	1.00	514.23	42.89	0.00	258.20	1.00	0.00	0.01	5.98	0.29	0.28	184.52	0.08	17.89	29.78	0.10
2019	**Wet**	HSM	Bissau	Reservoir outlet	8.12	29.46	0.25	1.00	514.03	42.78	0.00	258.48	1.00	0.00	0.01	6.02	0.29	0.29	185.21	0.08	17.49	30.00	0.10
2019	**Wet**	HSM	Bissau	Reservoir outlet	8.15	30.12	0.25	1.00	514.89	43.00	0.00	259.31	1.00	0.00	0.01	6.00	0.29	0.29	185.36	0.08	18.14	29.79	0.10
2019	**Wet**	HSM	Bissau	Reservoir outlet	8.15	29.68	0.25	1.00	514.79	42.69	0.00	259.12	1.00	0.00	0.01	5.99	0.29	0.29	185.00	0.08	18.25	30.00	0.10
2019	**Wet**	QJ	Bissau	Tap	7.95	29.18	0.22	3.00	496.00	26.90	0.00	230.00	1.00	0.01	0.04	8.00	0.24	0.24	205.00	0.08	15.00	60.00	0.12
2019	**Wet**	QJ	Bissau	Tap	8.00	30.00	0.22	2.99	494.30	27.01	0.00	228.90	0.99	0.01	0.04	8.00	0.24	0.24	204.20	0.08	14.87	60.00	0.12
2019	**Wet**	QJ	Bissau	Tap	8.21	29.12	0.22	3.01	495.85	26.79	0.00	229.64	0.98	0.01	0.04	8.00	0.24	0.23	204.78	0.08	15.02	59.89	0.12
2019	**Wet**	QJ	Bissau	Tap	8.12	29.32	0.22	3.00	495.46	27.00	0.00	230.14	1.00	0.01	0.04	8.00	0.24	0.24	205.75	0.08	15.00	60.02	0.12
2019	**Wet**	QJ	Bissau	Tap	7.99	29.24	0.22	2.95	494.89	27.08	0.00	230.85	1.02	0.01	0.04	8.00	0.24	0.25	205.36	0.08	15.40	60.49	0.12
2019	**Wet**	QJ	Bissau	Tap	7.90	30.01	0.22	3.12	496.05	26.89	0.00	230.45	1.03	0.01	0.04	8.00	0.24	0.24	204.98	0.08	14.79	60.21	0.12

Table S2: Annex 2 The raw data used for microbiological analysis. Footnote references is added below table.

Date	Season	Location	Sampling point	Coliforms	*Escherichia coli*	Enterococcus	aerobic mesophilic microorganisms	*Vibrio Cholerae*	*Vibrio Parahaemolyticus*	*Vibrio vulnificus*
2019	**Dry**	H3A	Hole	0.00	0.00	0.00	24.00	0.00	0.00	0.00
2019	**Dry**	H3A	Hole	0.00	0.00	0.00	26.00	0.00	0.00	0.00
2019	**Dry**	H3A	Hole	0.00	0.00	0.00	27.00	0.00	0.00	0.00
2019	**Dry**	H3A	Hole	0.00	0.00	0.00	28.00	0.00	0.00	0.00
2019	**Dry**	H3A	Hole	0.00	0.00	0.00	21.00	0.00	0.00	0.00
2019	**Dry**	H3A	Hole	0.00	0.00	0.00	26.00	0.00	0.00	0.00
2019	**Dry**	H3A	Tap	2.00	0.00	0.00	72.00	0.00	0.00	0.00
2019	**Dry**	H3A	Tap	2.00	0.00	0.00	83.00	0.00	0.00	0.00
2019	**Dry**	H3A	Tap	1.00	0.00	0.00	71.00	0.00	0.00	0.00
2019	**Dry**	H3A	Tap	2.00	0.00	0.00	69.00	0.00	0.00	0.00
2019	**Dry**	H3A	Tap	3.00	0.00	0.00	86.00	0.00	0.00	0.00
2019	**Dry**	H3A	Tap	3.00	0.00	0.00	81.00	0.00	0.00	0.00
2019	**Dry**	H3A	Fountain	5.00	0.00	0.00	70.00	0.00	0.00	0.00
2019	**Dry**	H3A	Fountain	4.00	0.00	0.00	61.00	0.00	0.00	0.00
2019	**Dry**	H3A	Fountain	6.00	0.00	0.00	60.00	0.00	0.00	0.00
2019	**Dry**	H3A	Fountain	5.00	0.00	0.00	65.00	0.00	0.00	0.00
2019	**Dry**	H3A	Fountain	5.00	0.00	0.00	56.00	0.00	0.00	0.00
2019	**Dry**	H3A	Fountain	4.00	0.00	0.00	68.00	0.00	0.00	0.00
2019	**Dry**	HSE	Hole	0.00	0.00	0.00	148.00	0.00	0.00	0.00
2019	**Dry**	HSE	Hole	0.00	0.00	0.00	100.00	0.00	0.00	0.00
2019	**Dry**	HSE	Hole	0.00	0.00	0.00	99.00	0.00	0.00	0.00
2019	**Dry**	HSE	Hole	0.00	0.00	0.00	102.00	0.00	0.00	0.00
2019	**Dry**	HSE	Hole	0.00	0.00	0.00	89.00	0.00	0.00	0.00
2019	**Dry**	HSE	Hole	0.00	0.00	0.00	95.00	0.00	0.00	0.00
2019	**Dry**	HSE	Reservoir outlet	3.00	0.00	0.00	198.00	0.00	0.00	0.00
2019	**Dry**	HSE	Reservoir outlet	1.00	0.00	0.00	128.00	0.00	0.00	0.00
2019	**Dry**	HSE	Reservoir outlet	2.00	0.00	0.00	125.00	0.00	0.00	0.00
2019	**Dry**	HSE	Reservoir outlet	2.00	0.00	0.00	132.00	0.00	0.00	0.00
2019	**Dry**	HSE	Reservoir outlet	2.00	0.00	0.00	124.00	0.00	0.00	0.00
2019	**Dry**	HSE	Reservoir outlet	3.00	0.00	0.00	98.00	0.00	0.00	0.00

2019	**Dry**	HSE	Fountain	7.00	1.00	0.00	200.00	0.00	0.00	0.00
2019	**Dry**	HSE	Fountain	5.00	0.00	0.00	202.00	0.00	0.00	0.00
2019	**Dry**	HSE	Fountain	4.00	2.00	0.00	189.00	0.00	0.00	0.00
2019	**Dry**	HSE	Fountain	6.00	1.00	0.00	192.00	0.00	0.00	0.00
2019	**Dry**	HSE	Fountain	5.00	1.00	0.00	201.00	0.00	0.00	0.00
2019	**Dry**	HSE	Fountain	7.00	1.00	0.00	189.00	0.00	0.00	0.00
2019	**Dry**	BLELA	Shallow wells	90.00	70.00	23.00	252.00	0.00	0.00	0.00
2019	**Dry**	BLELA	Shallow wells	70.00	29.00	21.00	250.00	0.00	0.00	0.00
2019	**Dry**	BLELA	Shallow wells	81.00	31.00	24.00	270.00	0.00	0.00	0.00
2019	**Dry**	BLELA	Shallow wells	78.00	32.00	27.00	258.00	0.00	0.00	0.00
2019	**Dry**	BLELA	Shallow wells	91.00	28.00	31.00	281.00	0.00	0.00	0.00
2019	**Dry**	BLELA	Shallow wells	89.00	30.00	35.00	275.00	0.00	0.00	0.00
2019	**Dry**	INTOZINHO	Shallow wells	120.00	80.00	14.00	280.00	0.00	0.00	0.00
2019	**Dry**	INTOZINHO	Shallow wells	150.00	83.00	21.00	290.00	0.00	0.00	0.00
2019	**Dry**	INTOZINHO	Shallow wells	160.00	45.00	19.00	289.00	0.00	0.00	0.00
2019	**Dry**	INTOZINHO	Shallow wells	152.00	29.00	21.00	285.00	0.00	0.00	0.00
2019	**Dry**	INTOZINHO	Shallow wells	157.00	30.00	17.00	279.00	0.00	0.00	0.00
2019	**Dry**	INTOZINHO	Shallow wells	165.00	33.00	20.00	287.00	0.00	0.00	0.00
2019	**Dry**	BOR	Shallow wells	39.00	5.00	17.00	152.00	0.00	0.00	0.00
2019	**Dry**	BOR	Shallow wells	29.00	2.00	11.00	164.00	0.00	0.00	0.00
2019	**Dry**	BOR	Shallow wells	30.00	2.00	10.00	160.00	0.00	0.00	0.00
2019	**Dry**	BOR	Shallow wells	31.00	1.00	12.00	158.00	0.00	0.00	0.00
2019	**Dry**	BOR	Shallow wells	32.00	2.00	14.00	164.00	0.00	0.00	0.00
2019	**Dry**	BOR	Shallow wells	28.00	2.00	15.00	114.00	0.00	0.00	0.00
2019	**Dry**	BOR	Shallow wells	30.00	19.00	19.00	90.00	0.00	0.00	0.00
2019	**Dry**	BOR	Shallow wells	35.00	25.00	10.00	312.00	0.00	0.00	0.00
2019	**Dry**	BOR	Shallow wells	29.00	20.00	8.00	308.00	0.00	0.00	0.00
2019	**Dry**	BOR	Shallow wells	34.00	16.00	11.00	280.00	0.00	0.00	0.00
2019	**Dry**	BOR	Shallow wells	36.00	21.00	7.00	302.00	0.00	0.00	0.00
2019	**Dry**	BOR	Shallow wells	36.00	21.00	10.00	304.00	0.00	0.00	0.00
2019	**Dry**	QUINHAMEL	Tubewell	28.00	1.00	4.00	78.00	0.00	0.00	0.00
2019	**Dry**	QUINHAMEL	Tubewell	21.00	1.00	2.00	80.00	0.00	0.00	0.00
2019	**Dry**	QUINHAMEL	Tubewell	23.00	1.00	1.00	69.00	0.00	0.00	0.00

2019	**Dry**	QUINHAMEL	Tubewell	24.00	1.00	1.00	75.00	0.00	0.00	0.00
2019	**Dry**	QUINHAMEL	Tubewell	20.00	2.00	1.00	85.00	0.00	0.00	0.00
2019	**Dry**	QUINHAMEL	Tubewell	18.00	2.00	0.00	82.00	0.00	0.00	0.00
2019	**Dry**	QUINHAMEL	Tubewell	35.00	2.00	2.00	70.00	0.00	0.00	0.00
2019	**Dry**	QUINHAMEL	Tubewell	21.00	1.00	1.00	60.00	0.00	0.00	0.00
2019	**Dry**	QUINHAMEL	Tubewell	19.00	1.00	1.00	58.00	0.00	0.00	0.00
2019	**Dry**	QUINHAMEL	Tubewell	23.00	1.00	0.00	62.00	0.00	0.00	0.00
2019	**Dry**	QUINHAMEL	Tubewell	24.00	2.00	0.00	59.00	0.00	0.00	0.00
2019	**Dry**	QUINHAMEL	Tubewell	20.00	1.00	1.00	61.00	0.00	0.00	0.00
2019	**Dry**	QUINHAMEL	Tubewell	29.00	2.00	3.00	41.00	0.00	0.00	0.00
2019	**Dry**	QUINHAMEL	Tubewell	39.00	2.00	2.00	52.00	0.00	0.00	0.00
2019	**Dry**	QUINHAMEL	Tubewell	40.00	2.00	2.00	60.00	0.00	0.00	0.00
2019	**Dry**	QUINHAMEL	Tubewell	38.00	2.00	1.00	78.00	0.00	0.00	0.00
2019	**Dry**	QUINHAMEL	Tubewell	41.00	3.00	0.00	85.00	0.00	0.00	0.00
2019	**Dry**	QUINHAMEL	Tubewell	37.00	4.00	0.00	92.00	0.00	0.00	0.00
2019	**Dry**	BAGDAD	Tubewell	16.00	2.00	0.00	134.00	0.00	0.00	0.00
2019	**Dry**	BAGDAD	Tubewell	19.00	1.00	0.00	128.00	0.00	0.00	0.00
2019	**Dry**	BAGDAD	Tubewell	31.00	2.00	1.00	130.00	0.00	0.00	0.00
2019	**Dry**	BAGDAD	Tubewell	21.00	3.00	0.00	135.00	0.00	0.00	0.00
2019	**Dry**	BAGDAD	Tubewell	32.00	4.00	1.00	129.00	0.00	0.00	0.00
2019	**Dry**	BAGDAD	Tubewell	29.00	2.00	0.00	140.00	0.00	0.00	0.00
2019	**Dry**	QUINHAMEL	Tubewell	12.00	2.00	1.00	140.00	0.00	0.00	0.00
2019	**Dry**	QUINHAMEL	Tubewell	11.00	1.00	1.00	124.00	0.00	0.00	0.00
2019	**Dry**	QUINHAMEL	Tubewell	15.00	1.00	1.00	130.00	0.00	0.00	0.00
2019	**Dry**	QUINHAMEL	Tubewell	12.00	0.00	1.00	136.00	0.00	0.00	0.00
2019	**Dry**	QUINHAMEL	Tubewell	16.00	2.00	0.00	140.00	0.00	0.00	0.00
2019	**Dry**	QUINHAMEL	Tubewell	11.00	2.00	1.00	131.00	0.00	0.00	0.00
2019	**Dry**	BANDIM	Tap	8.00	1.00	0.00	120.00	0.00	0.00	0.00
2019	**Dry**	BANDIM	Tap	7.00	1.00	0.00	122.00	0.00	0.00	0.00
2019	**Dry**	BANDIM	Tap	8.00	0.00	0.00	95.00	0.00	0.00	0.00
2019	**Dry**	BANDIM	Tap	6.00	1.00	0.00	96.00	0.00	0.00	0.00
2019	**Dry**	BANDIM	Tap	9.00	0.00	0.00	96.00	0.00	0.00	0.00
2019	**Dry**	BANDIM	Tap	9.00	0.00	0.00	94.00	0.00	0.00	0.00

2019	**Dry**	BANDIM	Fountain	12.00	0.00	0.00	104.00	0.00	0.00	0.00
2019	**Dry**	BANDIM	Fountain	9.00	1.00	0.00	107.00	0.00	0.00	0.00
2019	**Dry**	BANDIM	Fountain	8.00	1.00	0.00	109.00	0.00	0.00	0.00
2019	**Dry**	BANDIM	Fountain	10.00	0.00	0.00	105.00	0.00	0.00	0.00
2019	**Dry**	BANDIM	Fountain	9.00	1.00	0.00	112.00	0.00	0.00	0.00
2019	**Dry**	BANDIM	Fountain	7.00	1.00	0.00	110.00	0.00	0.00	0.00
2019	**Dry**	BANDIM	Hole	0.00	0.00	0.00	23.00	0.00	0.00	0.00
2019	**Dry**	BANDIM	Hole	0.00	0.00	0.00	26.00	0.00	0.00	0.00
2019	**Dry**	BANDIM	Hole	0.00	0.00	0.00	30.00	0.00	0.00	0.00
2019	**Dry**	BANDIM	Hole	0.00	0.00	0.00	35.00	0.00	0.00	0.00
2019	**Dry**	BANDIM	Hole	0.00	0.00	0.00	26.00	0.00	0.00	0.00
2019	**Dry**	BANDIM	Hole	0.00	0.00	0.00	28.00	0.00	0.00	0.00
2019	**Dry**	BANDIM	Reservoir outlet	8.00	2.00	0.00	95.00	0.00	0.00	0.00
2019	**Dry**	BANDIM	Reservoir outlet	10.00	0.00	0.00	89.00	0.00	0.00	0.00
2019	**Dry**	BANDIM	Reservoir outlet	11.00	0.00	0.00	90.00	0.00	0.00	0.00
2019	**Dry**	BANDIM	Reservoir outlet	12.00	1.00	0.00	93.00	0.00	0.00	0.00
2019	**Dry**	BANDIM	Reservoir outlet	15.00	1.00	0.00	87.00	0.00	0.00	0.00
2019	**Dry**	BANDIM	Reservoir outlet	14.00	2.00	0.00	84.00	0.00	0.00	0.00
2019	**Dry**	HSM	Fountain	10.00	0.00	0.00	260.00	0.00	0.00	0.00
2019	**Dry**	HSM	Fountain	10.00	0.00	0.00	280.00	0.00	0.00	0.00
2019	**Dry**	HSM	Fountain	11.00	2.00	0.00	286.00	0.00	0.00	0.00
2019	**Dry**	HSM	Fountain	10.00	1.00	0.00	280.00	0.00	0.00	0.00
2019	**Dry**	HSM	Fountain	9.00	1.00	0.00	250.00	0.00	0.00	0.00
2019	**Dry**	HSM	Fountain	12.00	1.00	0.00	198.00	0.00	0.00	0.00
2019	**Dry**	HSM	Reservoir outlet	4.00	1.00	0.00	201.00	0.00	0.00	0.00
2019	**Dry**	HSM	Reservoir outlet	5.00	0.00	0.00	198.00	0.00	0.00	0.00
2019	**Dry**	HSM	Reservoir outlet	6.00	1.00	0.00	189.00	0.00	0.00	0.00
2019	**Dry**	HSM	Reservoir outlet	4.00	1.00	0.00	186.00	0.00	0.00	0.00
2019	**Dry**	HSM	Reservoir outlet	5.00	2.00	0.00	200.00	0.00	0.00	0.00
2019	**Dry**	HSM	Reservoir outlet	7.00	2.00	0.00	182.00	0.00	0.00	0.00
2019	**Dry**	QJ	Tap	15.00	3.00	0.00	222.00	0.00	0.00	0.00
2019	**Dry**	QJ	Tap	12.00	1.00	0.00	200.00	0.00	0.00	0.00
2019	**Dry**	QJ	Tap	17.00	1.00	0.00	189.00	0.00	0.00	0.00

2019	**Dry**	QJ	Tap	21.00	0.00	0.00	201.00	0.00	0.00	0.00
2019	**Dry**	QJ	Tap	24.00	2.00	0.00	158.00	0.00	0.00	0.00
2019	**Dry**	QJ	Tap	16.00	1.00	0.00	216.00	0.00	0.00	0.00
2019	**Dry**	H3A	Hole	0.00	0.00	0.00	60.00	0.00	0.00	0.00
2019	**Wet**	H3A	Hole	0.00	0.00	0.00	64.00	0.00	0.00	0.00
2019	**Wet**	H3A	Hole	0.00	0.00	0.00	70.00	0.00	0.00	0.00
2019	**Wet**	H3A	Hole	0.00	0.00	0.00	65.00	0.00	0.00	0.00
2019	**Wet**	H3A	Hole	0.00	0.00	0.00	62.00	0.00	0.00	0.00
2019	**Wet**	H3A	Hole	0.00	0.00	0.00	65.00	0.00	0.00	0.00
2019	**Wet**	H3A	Tap	4.00	0.00	0.00	304.00	0.00	0.00	0.00
2019	**Wet**	H3A	Tap	4.00	0.00	0.00	308.00	0.00	0.00	0.00
2019	**Wet**	H3A	Tap	3.00	0.00	0.00	250.00	0.00	0.00	0.00
2019	**Wet**	H3A	Tap	3.00	0.00	0.00	296.00	0.00	0.00	0.00
2019	**Wet**	H3A	Tap	5.00	0.00	0.00	302.00	0.00	0.00	0.00
2019	**Wet**	H3A	Tap	3.00	0.00	0.00	320.00	0.00	0.00	0.00
2019	**Wet**	H3A	Fountain	10.00	3.00	0.00	312.00	0.00	0.00	0.00
2019	**Wet**	H3A	Fountain	7.00	2.00	0.00	292.00	0.00	0.00	0.00
2019	**Wet**	H3A	Fountain	9.00	2.00	0.00	290.00	0.00	0.00	0.00
2019	**Wet**	H3A	Fountain	12.00	1.00	0.00	300.00	0.00	0.00	0.00
2019	**Wet**	H3A	Fountain	8.00	1.00	0.00	301.00	0.00	0.00	0.00
2019	**Wet**	H3A	Fountain	9.00	0.00	0.00	306.00	0.00	0.00	0.00
2019	**Wet**	HSE	Hole	0.00	0.00	0.00	200.00	0.00	0.00	0.00
2019	**Wet**	HSE	Hole	0.00	0.00	0.00	180.00	0.00	0.00	0.00
2019	**Wet**	HSE	Hole	0.00	0.00	0.00	206.00	0.00	0.00	0.00
2019	**Wet**	HSE	Hole	0.00	0.00	0.00	159.00	0.00	0.00	0.00
2019	**Wet**	HSE	Hole	0.00	0.00	0.00	158.00	0.00	0.00	0.00
2019	**Wet**	HSE	Hole	0.00	0.00	0.00	201.00	0.00	0.00	0.00
2019	**Wet**	HSE	Reservoir outlet	4.00	1.00	0.00	212.00	0.00	0.00	0.00
2019	**Wet**	HSE	Reservoir outlet	3.00	1.00	0.00	215.00	0.00	0.00	0.00
2019	**Wet**	HSE	Reservoir outlet	5.00	0.00	0.00	218.00	0.00	0.00	0.00
2019	**Wet**	HSE	Reservoir outlet	6.00	0.00	0.00	209.00	0.00	0.00	0.00
2019	**Wet**	HSE	Reservoir outlet	7.00	2.00	0.00	216.00	0.00	0.00	0.00
2019	**Wet**	HSE	Reservoir outlet	8.00	2.00	0.00	218.00	0.00	0.00	0.00

2019	**Wet**	HSE	Fountain	8.00	2.00	0.00	296.00	0.00	0.00	0.00
2019	**Wet**	HSE	Fountain	7.00	0.00	0.00	292.00	0.00	0.00	0.00
2019	**Wet**	HSE	Fountain	10.00	0.00	0.00	189.00	0.00	0.00	0.00
2019	**Wet**	HSE	Fountain	12.00	2.00	0.00	203.00	0.00	0.00	0.00
2019	**Wet**	HSE	Fountain	15.00	3.00	0.00	221.00	0.00	0.00	0.00
2019	**Wet**	HSE	Fountain	18.00	2.00	0.00	240.00	0.00	0.00	0.00
2019	**Wet**	BLELA	Shallow wells	100.00	70.00	6.00	328.00	0.00	0.00	0.00
2019	**Wet**	BLELA	Shallow wells	96.00	75.00	4.00	336.00	0.00	0.00	0.00
2019	**Wet**	BLELA	Shallow wells	210.00	85.00	4.00	362.00	0.00	0.00	0.00
2019	**Wet**	BLELA	Shallow wells	183.00	82.00	3.00	360.00	0.00	0.00	0.00
2019	**Wet**	BLELA	Shallow wells	198.00	79.00	1.00	316.00	0.00	0.00	0.00
2019	**Wet**	BLELA	Shallow wells	204.00	91.00	5.00	316.00	0.00	0.00	0.00
2019	**Wet**	INTOZINHO	Shallow wells	336.00	120.00	3.00	344.00	0.00	0.00	0.00
2019	**Wet**	INTOZINHO	Shallow wells	324.00	154.00	2.00	324.00	0.00	0.00	0.00
2019	**Wet**	INTOZINHO	Shallow wells	300.00	150.00	1.00	336.00	0.00	0.00	0.00
2019	**Wet**	INTOZINHO	Shallow wells	304.00	152.00	2.00	340.00	0.00	0.00	0.00
2019	**Wet**	INTOZINHO	Shallow wells	308.00	161.00	2.00	312.00	0.00	0.00	0.00
2019	**Wet**	INTOZINHO	Shallow wells	308.00	159.00	2.00	320.00	0.00	0.00	0.00
2019	**Wet**	BOR	Shallow wells	43.00	7.00	1.00	324.00	0.00	0.00	0.00
2019	**Wet**	BOR	Shallow wells	39.00	3.00	2.00	336.00	0.00	0.00	0.00
2019	**Wet**	BOR	Shallow wells	41.00	2.00	1.00	340.00	0.00	0.00	0.00
2019	**Wet**	BOR	Shallow wells	52.00	3.00	1.00	336.00	0.00	0.00	0.00
2019	**Wet**	BOR	Shallow wells	50.00	1.00	2.00	320.00	0.00	0.00	0.00
2019	**Wet**	BOR	Shallow wells	53.00	3.00	1.00	324.00	0.00	0.00	0.00
2019	**Wet**	BOR	Shallow wells	40.00	37.00	1.00	328.00	0.00	0.00	0.00
2019	**Wet**	BOR	Shallow wells	38.00	9.00	1.00	312.00	0.00	0.00	0.00
2019	**Wet**	BOR	Shallow wells	48.00	5.00	1.00	356.00	0.00	0.00	0.00
2019	**Wet**	BOR	Shallow wells	52.00	6.00	0.00	350.00	0.00	0.00	0.00
2019	**Wet**	BOR	Shallow wells	39.00	7.00	1.00	356.00	0.00	0.00	0.00
2019	**Wet**	BOR	Shallow wells	48.00	9.00	2.00	354.00	0.00	0.00	0.00
2019	**Wet**	QUINHAMEL	Tubewell	25.00	1.00	0.00	60.00	0.00	0.00	0.00
2019	**Wet**	QUINHAMEL	Tubewell	35.00	1.00	0.00	52.00	0.00	0.00	0.00
2019	**Wet**	QUINHAMEL	Tubewell	36.00	2.00	0.00	62.00	0.00	0.00	0.00

2019	**Wet**	QUINHAMEL	Tubewell	40.00	1.00	0.00	50.00	0.00	0.00	0.00
2019	**Wet**	QUINHAMEL	Tubewell	41.00	2.00	0.00	61.00	0.00	0.00	0.00
2019	**Wet**	QUINHAMEL	Tubewell	39.00	1.00	0.00	65.00	0.00	0.00	0.00
2019	**Wet**	QUINHAMEL	Tubewell	101.00	2.00	0.00	172.00	0.00	0.00	0.00
2019	**Wet**	QUINHAMEL	Tubewell	99.00	2.00	0.00	164.00	0.00	0.00	0.00
2019	**Wet**	QUINHAMEL	Tubewell	103.00	1.00	0.00	160.00	0.00	0.00	0.00
2019	**Wet**	QUINHAMEL	Tubewell	100.00	1.00	0.00	180.00	0.00	0.00	0.00
2019	**Wet**	QUINHAMEL	Tubewell	98.00	2.00	0.00	179.00	0.00	0.00	0.00
2019	**Wet**	QUINHAMEL	Tubewell	79.00	1.00	0.00	181.00	0.00	0.00	0.00
2019	**Wet**	QUINHAMEL	Tubewell	31.00	6.00	0.00	284.00	0.00	0.00	0.00
2019	**Wet**	QUINHAMEL	Tubewell	21.00	4.00	0.00	274.00	0.00	0.00	0.00
2019	**Wet**	QUINHAMEL	Tubewell	25.00	2.00	0.00	280.00	0.00	0.00	0.00
2019	**Wet**	QUINHAMEL	Tubewell	23.00	2.00	0.00	276.00	0.00	0.00	0.00
2019	**Wet**	QUINHAMEL	Tubewell	32.00	1.00	0.00	259.00	0.00	0.00	0.00
2019	**Wet**	QUINHAMEL	Tubewell	35.00	3.00	0.00	270.00	0.00	0.00	0.00
2019	**Wet**	BAGDAD	Tubewell	36.00	4.00	0.00	288.00	0.00	0.00	0.00
2019	**Wet**	BAGDAD	Tubewell	25.00	3.00	0.00	312.00	0.00	0.00	0.00
2019	**Wet**	BAGDAD	Tubewell	25.00	2.00	0.00	280.00	0.00	0.00	0.00
2019	**Wet**	BAGDAD	Tubewell	29.00	1.00	0.00	181.00	0.00	0.00	0.00
2019	**Wet**	BAGDAD	Tubewell	25.00	0.00	0.00	204.00	0.00	0.00	0.00
2019	**Wet**	BAGDAD	Tubewell	27.00	1.00	0.00	284.00	0.00	0.00	0.00
2019	**Wet**	QUINHAMEL	Tubewell	20.00	2.00	0.00	304.00	0.00	0.00	0.00
2019	**Wet**	QUINHAMEL	Tubewell	18.00	2.00	0.00	300.00	0.00	0.00	0.00
2019	**Wet**	QUINHAMEL	Tubewell	25.00	0.00	0.00	281.00	0.00	0.00	0.00
2019	**Wet**	QUINHAMEL	Tubewell	22.00	1.00	0.00	288.00	0.00	0.00	0.00
2019	**Wet**	QUINHAMEL	Tubewell	27.00	2.00	0.00	270.00	0.00	0.00	0.00
2019	**Wet**	QUINHAMEL	Tubewell	27.00	2.00	0.00	259.00	0.00	0.00	0.00
2019	**Wet**	BANDIM	Tap	10.00	2.00	0.00	324.00	0.00	0.00	0.00
2019	**Wet**	BANDIM	Tap	8.00	1.00	0.00	320.00	0.00	0.00	0.00
2019	**Wet**	BANDIM	Tap	12.00	1.00	0.00	260.00	0.00	0.00	0.00
2019	**Wet**	BANDIM	Tap	15.00	1.00	0.00	270.00	0.00	0.00	0.00
2019	**Wet**	BANDIM	Tap	16.00	1.00	0.00	304.00	0.00	0.00	0.00
2019	**Wet**	BANDIM	Tap	16.00	1.00	0.00	204.00	0.00	0.00	0.00

2019	**Wet**	BANDIM	Fountain	9.00	1.00	0.00	298.00	0.00	0.00	0.00
2019	**Wet**	BANDIM	Fountain	7.00	1.00	0.00	292.00	0.00	0.00	0.00
2019	**Wet**	BANDIM	Fountain	15.00	1.00	0.00	305.00	0.00	0.00	0.00
2019	**Wet**	BANDIM	Fountain	18.00	1.00	0.00	289.00	0.00	0.00	0.00
2019	**Wet**	BANDIM	Fountain	18.00	1.00	0.00	301.00	0.00	0.00	0.00
2019	**Wet**	BANDIM	Fountain	19.00	1.00	0.00	304.00	0.00	0.00	0.00
2019	**Wet**	BANDIM	Hole	0.00	0.00	0.00	30.00	0.00	0.00	0.00
2019	**Wet**	BANDIM	Hole	0.00	0.00	0.00	35.00	0.00	0.00	0.00
2019	**Wet**	BANDIM	Hole	0.00	0.00	0.00	40.00	0.00	0.00	0.00
2019	**Wet**	BANDIM	Hole	0.00	0.00	0.00	42.00	0.00	0.00	0.00
2019	**Wet**	BANDIM	Hole	0.00	0.00	0.00	47.00	0.00	0.00	0.00
2019	**Wet**	BANDIM	Hole	0.00	0.00	0.00	50.00	0.00	0.00	0.00
2019	**Wet**	BANDIM	Reservoir outlet	15.00	0.00	0.00	280.00	0.00	0.00	0.00
2019	**Wet**	BANDIM	Reservoir outlet	13.00	0.00	0.00	260.00	0.00	0.00	0.00
2019	**Wet**	BANDIM	Reservoir outlet	16.00	1.00	0.00	282.00	0.00	0.00	0.00
2019	**Wet**	BANDIM	Reservoir outlet	14.00	1.00	0.00	280.00	0.00	0.00	0.00
2019	**Wet**	BANDIM	Reservoir outlet	12.00	1.00	0.00	186.00	0.00	0.00	0.00
2019	**Wet**	BANDIM	Reservoir outlet	17.00	1.00	0.00	196.00	0.00	0.00	0.00
2019	**Wet**	HSM	Fountain	12.00	0.00	0.00	280.00	0.00	0.00	0.00
2019	**Wet**	HSM	Fountain	15.00	1.00	0.00	300.00	0.00	0.00	0.00
2019	**Wet**	HSM	Fountain	17.00	0.00	0.00	280.00	0.00	0.00	0.00
2019	**Wet**	HSM	Fountain	18.00	0.00	0.00	282.00	0.00	0.00	0.00
2019	**Wet**	HSM	Fountain	14.00	1.00	0.00	302.00	0.00	0.00	0.00
2019	**Wet**	HSM	Fountain	17.00	1.00	0.00	300.00	0.00	0.00	0.00
2019	**Wet**	HSM	Reservoir outlet	6.00	2.00	0.00	289.00	0.00	0.00	0.00
2019	**Wet**	HSM	Reservoir outlet	7.00	2.00	0.00	291.00	0.00	0.00	0.00
2019	**Wet**	HSM	Reservoir outlet	8.00	1.00	0.00	287.00	0.00	0.00	0.00
2019	**Wet**	HSM	Reservoir outlet	7.00	1.00	0.00	284.00	0.00	0.00	0.00
2019	**Wet**	HSM	Reservoir outlet	6.00	1.00	0.00	196.00	0.00	0.00	0.00
2019	**Wet**	HSM	Reservoir outlet	8.00	1.00	0.00	201.00	0.00	0.00	0.00
2019	**Wet**	QJ	Tap	17.00	2.00	0.00	260.00	0.00	0.00	0.00
2019	**Wet**	QJ	Tap	19.00	2.00	0.00	244.00	0.00	0.00	0.00
2019	**Wet**	QJ	Tap	21.00	1.00	0.00	244.00	0.00	0.00	0.00

2019	**Wet**	QJ	Tap	25.00	1.00	0.00	189.00	0.00	0.00	0.00
2019	**Wet**	QJ	Tap	28.00	1.00	0.00	196.00	0.00	0.00	0.00
2019	**Wet**	QJ	Tap	24.00	1.00	0.00	260.00	0.00	0.00	0.00
